# Supplementary material for: Effect of Extraction Solvent and Temperature on Polyphenol Profiles, Antioxidant and Anti-Inflammatory Effects of Red Grape Skin By-Product
Source: Molecules. 2021 Sep 7;26(18):5454. doi: 10.3390/molecules26185454 (PMC8471016; doi:10.3390/molecules26185454)
Supplement: Supplementary file 1 [file molecules-26-05454-s001.zip › molecules-1332197-supplementary.pdf]

molecules

MDPI

Supplementary Materials

Effect of Extraction Solvent and Temperature on Polyphenol Profiles, Antioxidant and Anti-Inflammatory Effects of Red Grape Skin By-Product

Giovanna Baron [1,*], Giulio Ferrario [1], Cristina Marinello [1], Marina Carini [1], Paolo Morazzoni [2] and Giancarlo Aldini [1]

[1] Department of Pharmaceutical Sciences (DISFARM), Università degli Studi di Milano, Via Mangiagalli 25, 20133 Milan, Italy; giulio.ferrario1@unimi.it (G.F.); cristina.marinello@unimi.it (C.M.); marina.carini@unimi.it (M.C.); giancarlo.aldini@unimi.it (G.A.)

[2] Distillerie Umberto Bonollo S.p.A, Divisione Nutraceutica, 35035 Mestrino (PD), Italy; paolo.morazzoni@bonollo.it

* Correspondence: giovanna.baron@unimi.it

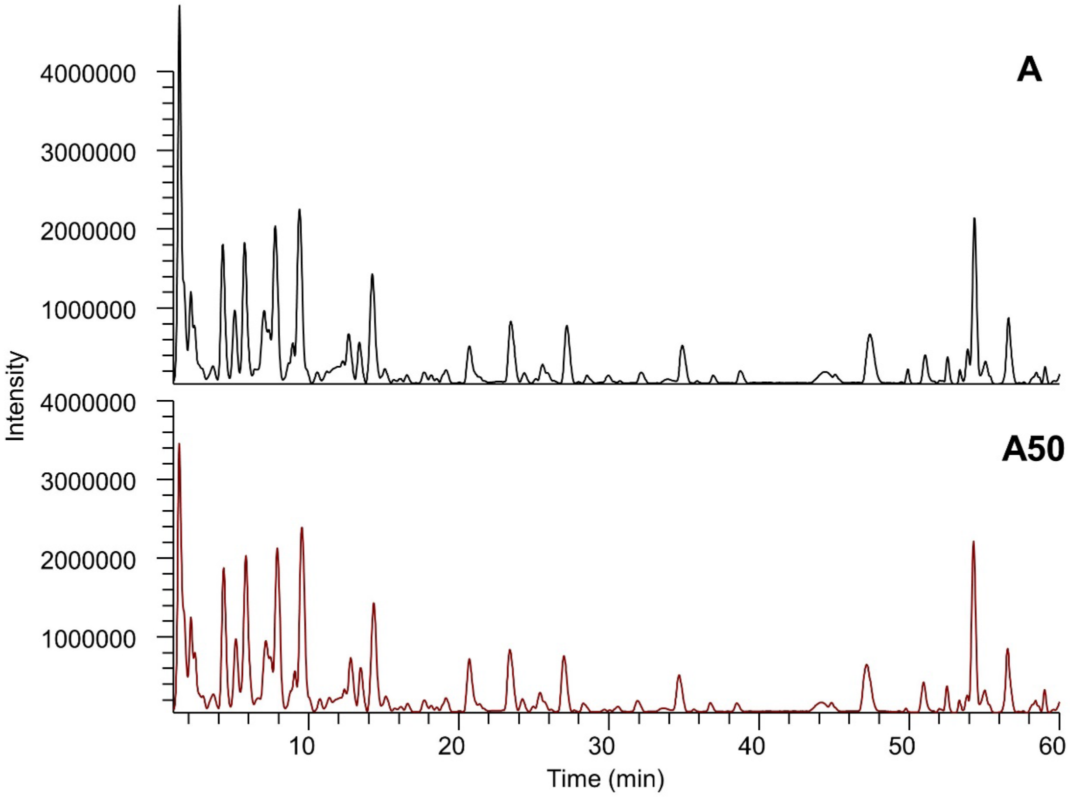

Figure S1. LC-ESI-(-)-MS total ion currents (TICs) in negative ion mode of the hydroalcoholic extracts : A) EtOH-H_2O 70:30 (% *v*/*v*) at room temperature for 24 h, extract A; A50) EtOH-H_2O 70:30 (% *v*/*v*) at 50 °C for 1 h followed by 24 h at room temperature, extract A50.

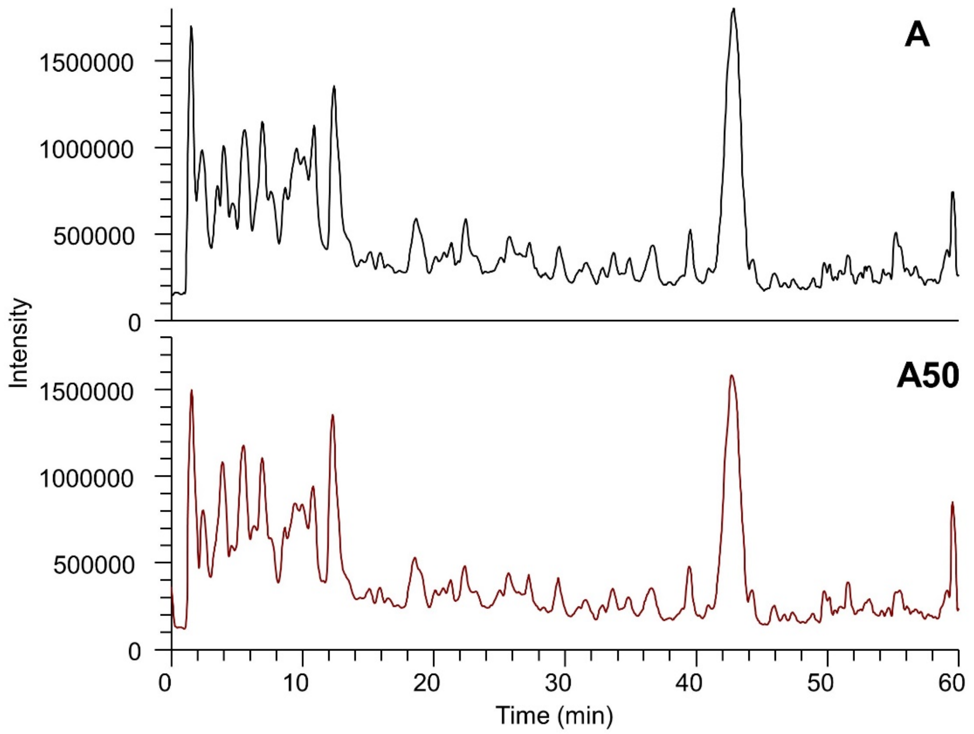

Figure S2. LC-ESI-(+)-MS total ion currents (TICs) in positive ion mode of the hydroalcoholic extracts : A) EtOH-H_2O 70:30 (% *v*/*v*) at room temperature for 24 h, extract A; A50) EtOH-H_2O 70:30 (% *v*/*v*) at 50 °C for 1 h followed by 24 h at room temperature, extract A50.
